# The necessity of gestational vitamin D supplementation depends on ambient temperature: concern for infant vitamin D status

**DOI:** 10.3389/fnut.2025.1541427

**Published:** 2025-01-28

**Authors:** Wanjun Yin, Lei Zhang, Peng Zhu

**Affiliations:** ^1^Joint Research Center of Occupational Medicine and Health, Institute of Grand Health, Hefei Comprehensive National Science Center, Anhui University of Science and Technology, Hefei, China; ^2^School of Public Health, Anhui University of Science and Technology, Hefei, China; ^3^Key Laboratory of Industrial Dust Prevention and Control, Occupational Safety and Health, Ministry of Education, Anhui University of Science and Technology, Huainan, China; ^4^MOE Key Laboratory of Population Health Across Life Cycle, Hefei, China; ^5^Department of Maternal, Child & Adolescent Health, School of Public Health, Anhui Medical University, Hefei, China

**Keywords:** pregnant women, vitamin D supplementation, vitamin D, ambient temperature, neonate

## Abstract

**Background:**

Given the ease of access to ambient temperature, it may be a more practical guide than the UVB index. However, the association between gestational temperature and vitamin D level in newborns remains unclear. Our study aims to explore this association and the necessity of maternal vitamin D supplementation when ambient temperature is less than a specific value.

**Methods:**

Based on a birth cohort study, we measured cord blood concentrations of 25(OH)D in 1419 neonates from January to September 2008 in Hefei, a new first-tier city in China. The daily mean temperature of Hefei was obtained from the China Meteorological Data Sharing Service System. Individual information on sociodemographic characteristics, perinatal health status, lifestyle, and birth outcomes was collected prospectively.

**Results:**

The best-fit relationship was observed in the regression model using a quadratic function to describe the association between the ambient temperature of the eighth gestational month (29–32 gestational weeks) and cord blood 25(OH)D concentrations (*R*^2^ = 0.358, *p* < 0.001). Ambient temperatures of 10 and 24.5°C were linked to the cutoff of vitamin D inadequacy (<50 nmol/L) and deficiency (<30 nmol/L) in cord blood, respectively. For maternal exposure to an ambient temperature of ≥24.5°C in the eighth gestational month, vitamin D supplementation during pregnancy failed to significantly enhance neonatal vitamin D concentrations. In contrast, for maternal exposure to ambient temperature of <10°C, maternal vitamin D supplementation was significantly associated with elevated 25(OH)D concentrations in cord blood.

**Conclusion:**

Gestational ambient temperature may be an ideal predictor for infant vitamin D status screening. Maternal exposure to an ambient temperature of less than 10°C is a critical index in the eighth gestational month, which may determine the onset of vitamin D supplementation.

## Introduction

Vitamin D deficiency during pregnancy and the neonatal period is a common global health concern (1, 2). Studies on pregnant women have reported a high prevalence of vitamin D deficiency in Colombia (23.9%) (3), France (41.5%) (4), and China (76.4%) (5). It has been confirmed that 25-hydroxyvitamin D [25(OH)D], the major circulating form of vitamin D, could readily traverse the hemochorial placenta (6). Fetal vitamin D concentrations are largely dependent on maternal vitamin D status (7). To date, guidelines concerning vitamin D screening in pregnant women have been conflicting (8–13). The Committee Opinion of the American College of Obstetricians and Gynecologists (8) and Australian Government antenatal care clinical practice guidelines (10) recommend screening only women at increased risk of vitamin D deficiency, including vegetarians, women with limited sun exposure, and women with darker skin (14). Hence, identifying pregnant women at increased risk of vitamin D deficiency is an important public health strategy.

The main source of vitamin D in most humans is the synthesis of 7-dehydrocholesterol in the skin upon exposure to ultraviolet B (UVB) solar radiation and its conversion to a measurable circulating metabolite, 25(OH)D, in the liver (15), which is used to determine individual vitamin D status (16). Thus, limited sunlight exposure may influence skin production of vitamin D, resulting in vitamin D deficiency. However, several factors influence the intensity and duration of exposure to UVB radiation, including geographic location, season, atmospheric conditions, and time spent outdoors (17). Notably, the ambient temperature is usually associated with both UVB radiation and its influencing factors. Although there is no direct causality between ambient temperature and vitamin D, given the access to ambient temperature, it may be a better practical predictor of individual vitamin D concentrations. However, to the best of our knowledge, the relationship between ambient temperature and individual vitamin D concentration has not been well established.

We hypothesized that maternal exposure to ambient temperature might be positively associated with fetal vitamin D concentrations and that cutaneous synthesis of previtamin D could be unavailable when the ambient temperature is greatly reduced (i.e., when the amount of solar ultraviolet radiation that reaches the earth’s surface is substantially reduced). This leaves the individual uniquely dependent on supplemental sources of vitamin D during periods when ambient temperature is below the threshold value. This study aimed to determine the implications of gestational ambient temperature on infant vitamin D status and to establish the necessity of maternal vitamin D supplementation when the ambient temperature is less than a threshold value. The significance of this study lies in its ability to reveal the effects of ambient temperature on vitamin D supplementation during pregnancy. If ambient temperature is shown to be a reliable predictor of vitamin D deficiency or supplementation needs, it could significantly impact public health strategies, especially in regions with seasonal temperature variations. The findings of this study provide new insights into how environmental factors, particularly temperature, should be considered when developing pregnancy-care guidelines. This could lead to more individualized recommendations for vitamin D supplementation based on the local climate, particularly during colder months when vitamin D synthesis is impaired owing to reduced UVB exposure. Furthermore, this study could lay the foundation for future research on the relationship between environmental factors and maternal vitamin D status, ultimately guiding more effective prenatal care policies to improve maternal and fetal health outcomes.

## Subjects and methods

### Study design

A total of 2,552 pregnant women with gestational ages between 30 and 34 weeks were recruited at the Hefei Maternal and Child Health Hospital (32°N latitude) between January and September 2008. The inclusion criteria for this study were age ≥ 18 years, absence of communication problems, and residents of Hefei city. The participants completed a structured questionnaire that included questions on their demographic characteristics and perinatal lifestyle through face-to-face interviews. After delivery, well-trained nurses collected the infant anthropometric details and cord blood samples. In this study, we restricted the study sample to full-term births. Women with premature births, stillbirths, birth defects, pregnancies with assisted reproductive technology, or multiple gestations were excluded to avoid confounding factors. Finally, we obtained available data from 1,419 mother-infant pairs with cord blood samples. Informed consent was obtained from each participant. The research protocol was approved by the Ethics Committee of Anhui Medical University (Approval Number: 2008020).

### Ambient temperature exposure

The daily mean temperature of Hefei was obtained from the China Meteorological Data Sharing Service System^1^. This system provides publicly available temperature data, including the daily average temperature of Hefei based on measurements collected from several meteorological stations across the city. These measurements were taken at a standard height of 2 meters above ground level, which is the standard method for collecting ambient temperature data. The daily mean temperature reflects the average temperature for each 24-h period. To estimate the ambient temperature exposure of each participant, we used the last menstrual period (LMP) date of each woman to calculate their gestational months (GM). The LMP was used to estimate gestational age as it provides a reliable reference point for determining the timing of pregnancy stages. For each participant, gestational months were divided into four-week intervals, with each GM consisting of approximately 4 weeks. Once gestational months were identified, we calculated the mean ambient temperature for each specific GM during pregnancy. This involved averaging daily temperature readings from the China Meteorological Data Sharing Service System for the corresponding weeks of each participant’s pregnancy. Daily temperature data were averaged over each gestational month to provide an estimate of the mean temperature for that month. In addition, ambient temperatures were categorized into three distinct ranges based on their potential impact on UVB radiation exposure and fetal vitamin D synthesis: <10°C, 10–24.4°C, and ≥24.5°C.

### Vitamin D supplementation assessment

When pregnant women first underwent prenatal checkups in obstetric clinics, they were advised to take 600 IU/d of vitamin D3 supplements during the second and third trimesters, according to the guidelines of the Institute of Medicine (IOM) (11). In the interview conducted between 32 and 34 gestational weeks, each participant was invited to respond to the two questions: “How long have you taken vitamin D during pregnancy?” and “What’s the daily dose of vitamin D supplementation you have taken during pregnancy?” However, most pregnant women are unable to report the exact dose of vitamin D supplementation. By collecting information on the brands of vitamin D supplements and their frequency of use, we found that the majority of pregnant women were taking commercial vitamin D at doses ranging from 300 IU to 600 IU per day. For analytical purposes, the duration of vitamin D supplementation was selected as the index of vitamin D supplementation and categorized as per the following prioritization: <2 months and ≥2 months.

### Cord blood 25(OH)D measurement

Cord blood samples were collected immediately after delivery. Plasma samples were centrifuged and promptly refrigerated at −4°C and then transferred to −80°C freezers within 12 h for long-term storage. Cord blood 25(OH)D concentrations were measured using commercial radioimmunoassay kits (DiaSorin Stillwater, MN, United States) by well-trained researchers at the Anhui Provincial Key Laboratory of Population Health and Aristogenics (5). The intra- and interassay coefficients of variation were 8.8 and 11.1%, respectively. According to the IOM classification (18), vitamin D status was defined as deficient [25(OH)D < 30 nmol/L] or inadequate [30 nmol/L ≤ 25(OH)D < 50 nmol/L].

### Covariates

Information on essential potential confounders was collected from medical records or interviews in this study. Maternal sociodemographic characteristics included age, education (≤9 and >9 years of completed schooling), and household income (<2000 and ≥2000 Yuan RMB/month). Perinatal health status included pre-pregnancy body mass index (BMI), gestational weight gain (GWG), pregnancy complications (including moderate or severe anemia, hypertension, diabetes mellitus, glandula thyreoidea disease, abnormal heart function, and intrahepatic cholestasis of pregnancy), and parity (nullipara or multipara). The pre-pregnancy lifestyle included daily outdoor time (<1 h/d vs. ≥1 h/d), maternal alcohol consumption, paternal smoking, and alcohol consumption for up to 6 months before pregnancy. The birth outcomes included gestational age, birth weight, and infant sex. Small for gestational age (SGA) was defined as birth weight < 10th percentiles of distribution for gestational age and infant gender (19). The birth seasons were designated as follows: spring (March, April, and May), summer (June, July, and August), fall (September, October, and November), and winter (December, January, and February).

### Statistical analysis

**Figure 1:**
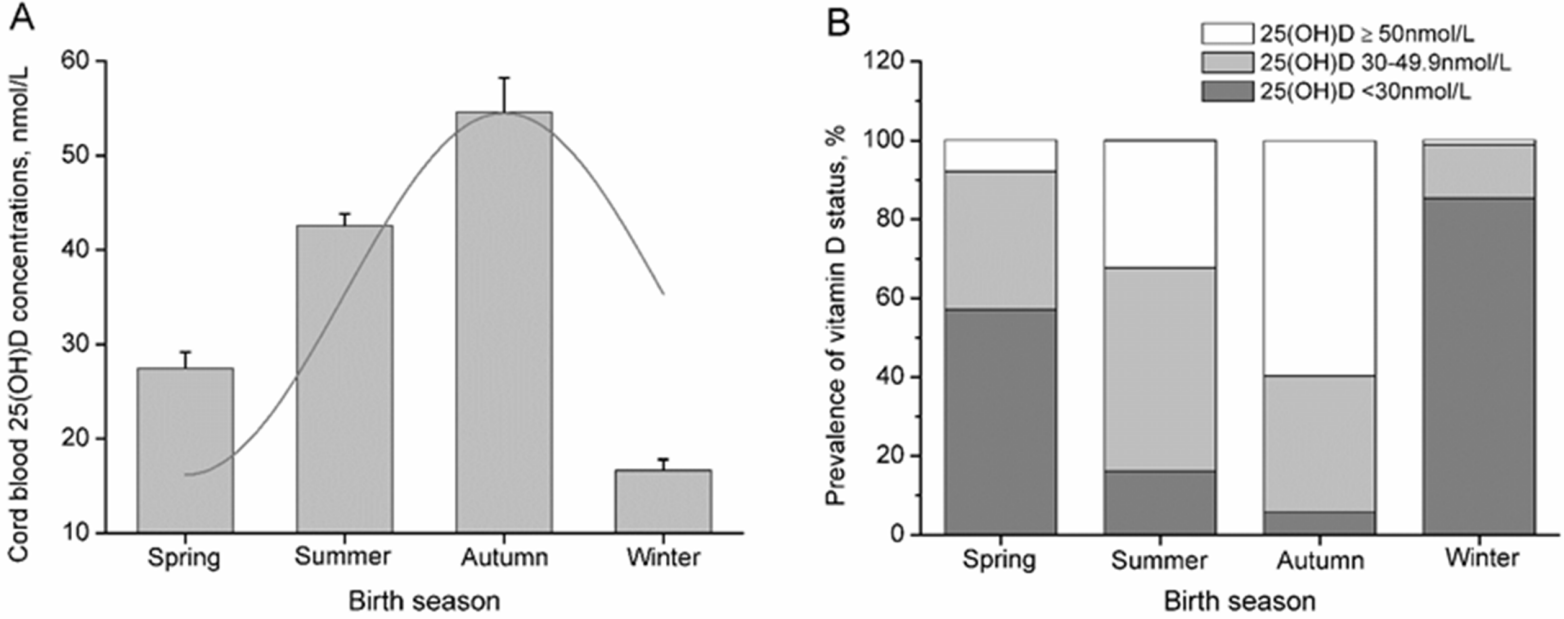
Cord blood vitamin D status across the birth season in pregnant women. The seasonality of cord blood 25(OH)D levels was estimated by fitting the data to the best-fitting linear model using a sinusoidal model **(A)**, and the bars represent the median and 95% CI **(A)** or percentage of subjects **(B)**.

Associations between the characteristics of mother-infant pairs and cord blood 25(OH)D levels were examined to determine potential covariates in subsequent analyses (Table 1). The trend in cord blood 25(OH)D concentrations across birth seasons was estimated using a sinusoidal model, and infant vitamin D status across birth seasons was examined using the chi-square test (Figure 1).

**Table 1:** Characteristics of pregnant women in relation to cord blood 25(OH)D.

Characteristics	Classify	Mean (SD) or *n* (%)	*β* (95% CI)	*p*-value
Sociodemographic characteristics				
Maternal age, y		27.68 (3.61)	0.04 (−0.25, 0.33)	0.796
Maternal education	<9 years	282 (19.9)	−0.71 (−3.35, 1.94)	0.600
	≥9 years	1,137 (80.1)	Ref	
Maternal income	<2000 yuan	207 (14.6)	−0.99 (−3.97, 2.00)	0.516
	≥ 2000 yuan	1,212 (85.4)	Ref	
Perinatal health status				
Pre-pregnancy BMI, kg/m^2^		20.15 (2.40)	−0.19 (−0.63, 0.25)	0.394
GWG, kg		16.90 (4.84)	−0.36 (−0.57, −0.14)	0.001
Multipara	Primipara	1,234 (87.0)	Ref	
	Multipara	185 (13.0)	−0.47 (−3.60, 2.66)	0.768
Pregnancy complications^1^	Yes	211 (14.9)	−3.27 (−6.23, −0.32)	0.030
	No	1,208 (85.1)	Ref	
Perinatal lifestyle^2^				
Maternal alcohol consumption	Any	216 (15.2)	−1.60 (−4.54, 1.33)	0.284
	No	1,203 (84.8)	Ref	
Paternal alcohol consumption	Any	1,137 (80.1)	3.42 (0.79, 6.06)	0.011
	No	282 (19.9)	Ref	
Paternal smoking^3^	Yes	317 (22.3)	0.75 (−1.78, 3.28)	0.561
	No	1,102 (77.7)	Ref	
Daily outdoor time	≥1 h/d	486 (34.2)	2.62 (0.44, 4.79)	0.018
	<1 h/d	933 (65.8)	Ref	
Vitamin D supplementation	≥2 months	691 (48.7)	2.66 (0.56, 4.77)	0.013
	<2 months	728 (51.3)	Ref	
Birth outcomes				
Infant gender	Female	667 (47.0)	−2.23 (−4.34, −0.13)	0.038
	Male	752 (53.0)	Ref	
Birth weight, g		3,420 (419)	0.001 (−0.003, 0.002)	0.801
SGA	Yes	122 (8.6)	−1.92 (−5.68, 1.84)	0.317
	No	1,297 (91.4)	Ref	

25(OH)D, 25-hydroxyvitamin D; BMI, body mass index; GWG, gestational weight gain; SGA, small for gestational age.

^1Complications of pregnancy included diabetes mellitus, hypertension, abnormal heart function, glandula thyreoidea disease, intrahepatic cholestasis of pregnancy, and moderate and severe anemia.^

^2Pre-pregnancy lifestyle means lifestyle during up to 6 months before pregnancy.^

^3Paternal smoking was defined as smoking more than six cigarettes daily.^

**Figure 2:**
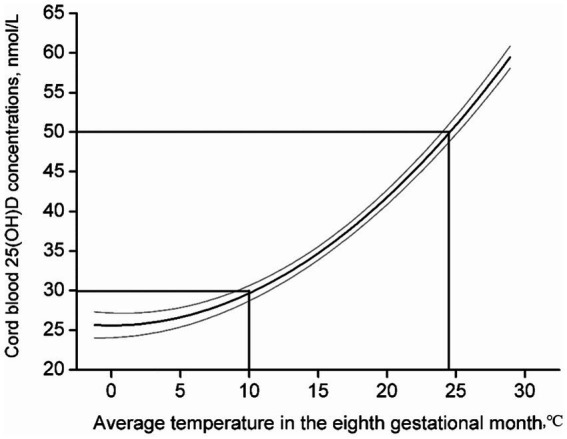
Relationship between ambient temperature in the eighth GM and cord blood 25(OH)D in pregnant women. The solid dark line represents the trend for changes in cord blood 25(OH)D mean values with increasing ambient temperature in the eighth GM, and the gray lines represent the 95% confidence interval.

To confirm the critical time when maternal exposure to ambient temperature influences cord blood 25(OH)D concentrations, linear and non-linear regression models (quadratic and cubic) were used to assess the relationships between the ambient temperature of each GM and cord blood 25(OH)D concentration, respectively. The best-fit relationship was observed in the eighth GM, with a quadratic function (Table 2). Consequently, the eighth GM was considered a critical time when maternal exposure to ambient temperature influenced cord blood 25(OH)D concentration in subsequent analyses. The regression model with the quadratic function suggested that ambient temperatures of 10°C and 24.5°C in the eighth GM were linked to the cutoff of vitamin D inadequacy and vitamin D deficiency in cord blood, respectively (Figure 2). Odds ratios (*OR*) and 95% confidence intervals (CI) of vitamin D deficiency and vitamin D inadequacy for maternal exposure to 10–24.4°C and <10°C in the eighth GM were generated using a multiple logistic regression model (Table 3).

**Table 2:** Linear, quadratic, and curve estimations for the associations between ambient temperature of gestation and cord blood 25(OH)D in pregnant women.

Ambient temperature	*R* ^2^	*F*	*p*
37–40 gestational weeks			
Linear	0.258	492.0	<0.001
Quadratic	0.270	261.9	<0.001
Cubic	0.271	243.3	<0.001
33–36 gestational weeks			
Linear	0.305	620.5	<0.001
Quadratic	0.329	346.5	<0.001
Cubic	0.328	344.3	<0.001
29–32 gestational weeks			
Linear	0.338	724.6	<0.001
Quadratic	0.358	394.0	<0.001
Cubic	0.358	392.4	<0.001
25–28 gestational weeks			
Linear	0.244	457.6	<0.001
Quadratic	0.271	262.5	<0.001
Cubic	0.273	260.4	<0.001
21–24 gestational weeks			
Linear	0.107	170.1	<0.001
Quadratic	0.152	126.8	<0.001
Cubic	0.154	114.6	<0.001

**Table 3:** Risk for ambient temperature in the eighth GM and maternal vitamin D supplementation on vitamin D deficiency or inadequacy in cord blood in pregnant women.

Predictor variables	%	Vitamin D deficiency [25(OH)D < 30 nmol/L]		%	Vitamin D inadequacy [25(OH)D < 50 nmol/L]	
		Unadjusted OR (95% CI)	Adjusted OR^1^ (95% CI)		Unadjusted OR (95% CI)	Adjusted OR^1^ (95% CI)
Temperature						
<10°C	68.1	24.72 (16.35, 37.37)^#^	24.60 (15.83, 38.21)^#^	94.5	17.65 (11.41, 27.29)^#^	16.58 (10.23, 26.87)^#^
10–24.4°C	25.9	4.05 (2.68, 6.13)^#^	4.01 (2.64, 6.09)^#^	76.5	3.35 (2.53, 4.44)^#^	3.50 (2.61, 4.69)^#^
≥24.5°C	7.9	1.0	1.0	49.2	1.0	1.0
VD supplementation						
≥2 months	33.1	0.82 (0.66, 1.02)*	0.70 (0.54, 0.91)**	72.5	0.77 (0.60, 0.98)**	0.66 (0.50, 0.87)**
<2 months	37.8	1.0	1.0	77.6	1.0	1.0

Adjusted for gestational weight gain, pregnancy complications (diabetes mellitus, hypertension, abnormal heart function, glandula thyreoidea disease, intrahepatic cholestasis of pregnancy, moderate and severe anemia), paternal alcohol consumption, daily outdoor time, and infant sex.

*p < 0.1, **p < 0.05, ^#^*p* < 0.01.

Stratified by the ambient temperature of the eighth GM, hierarchical multiple regression analyses were conducted to assess the relative contributions of ambient temperature and vitamin D supplementation during pregnancy to cord blood 25(OH)D concentration. For these analyses, we allowed potential covariates to enter the first blocks in a stepwise fashion, then forced the ambient temperature of the eighth GM into the equation and entered vitamin D supplementation (Table 4). All statistical analyses were performed using the Statistical Package for the Social Sciences (SPSS) statistical software (version 21.0).

**Table 4:** Regression analyses for predictor variables and cord blood 25(OH)D, stratified by ambient temperature in the eighth GM in pregnant women.

Predictor variables	<10°C (*n* = 489)					10–24.4°C (*n* = 540)					≥24.5°C (*n* = 390)				
	Unstandardized *β*	SE	Standardized *β*	*R* ^2^	*ΔR^2^*	Unstandardized *β*	SE	Standardized *β*	*R* ^2^	*ΔR^2^*	Unstandardized *β*	SE	Standardized *β*	*R* ^2^	*ΔR^2^*
Step 1				0.024					0.011					0.032	
Paternal alcohol consumption	1.37	1.45	0.04			1.97	1.75	0.05			4.13	2.67	0.08		
GWG	0.11	0.12	0.04			−0.13	0.15	−0.04			−0.31	0.21	−0.07		
Pregnancy complications	−1.00	1.57	−0.03			−2.98	1.84	−0.07			−3.40	3.20	−0.05		
Daily outdoor time	1.12	1.51	0.04			0.36	1.41	0.01			0.68	1.33	0.02		
Infant gender	−3.23	1.16	−0.13^#^			−0.42	1.40	−0.01			−1.09	2.07	−0.03		
Step 2				0.027	0.003				0.128	0.117	4.13	2.67	0.08	0.127	0.095
Paternal alcohol consumption	1.41	1.45	0.05			0.61	1.65	0.02			3.21	2.54	0.06		
GWG	0.1	0.12	0.04			−0.01	0.14	0.00			−0.28	0.2	−0.07		
Pregnancy complications	−0.97	1.57	−0.03			−2.16	1.73	−0.05			−4.73	3.05	−0.07		
Daily outdoor time	0.65	1.55	0.02			0.33	1.32	0.01			0.57	1.45	0.02		
Infant gender	−3.32	1.16	−0.13**			−0.53	1.31	−0.02			−0.67	1.97	−0.02		
Ambient temperature	0.24	0.2	0.06*			1.09	0.13	0.35^#^			4.36	0.65	0.31^#^		
Step 3				0.033	0.005				0.266	0.138				0.131	0.004
Paternal alcohol consumption	1.31	1.45	0.04			0.37	1.51	0.01			3.56	2.4	0.07		
GWG	0.11	0.12	0.04			−0.04	0.13	−0.01			−0.38	0.19	−0.09		
Pregnancy complications	−0.87	1.57	−0.03			−2.22	1.59	−0.05			−3.37	2.89	−0.05		
Daily outdoor time	0.67	1.76	−0.02			0.41	1.40	−0.01			0.27	1.25	0.01		
Infant gender	−3.42	1.16	−0.14^#^			−1.68	1.21	−0.05			0.31	1.87	0.01		
Ambient temperature	0.27	0.2	0.07			1.09	0.12	0.35^#^			3.65	0.62	0.26^#^		
Vitamin D supplementation	2.17	1.14	0.09**			4.65	1.30	0.14^#^			2.98	2.00	0.070		

GM, gestational month; GWG, gestational weight gain.

*p < 0.1, **p < 0.05, ^#^*p* < 0.01.

## Results

### Effect of temperature on vitamin D supplementation

Attrition analyses revealed no significant difference in the distributions of demographic characteristics, perinatal health status, lifestyle, and birth outcomes between participants and non-participants. Table 1 shows the characteristics of mother-infant pairs in this study and their association with cord blood 25(OH)D concentrations. Cord blood 25(OH)D concentrations were suggested to be significantly related to GWG, pregnancy complications, paternal alcohol consumption, daily outdoor time, and infant sex, which were considered potential covariates in subsequent analyses.

Cord blood 25(OH)D concentrations showed a seasonal distribution with a mean concentration of 39.52 nmol/L and SD of 20.23 nmol/L. The maximum fitted concentrations of cord blood 25(OH)D were observed in neonates born in autumn, reaching their nadir in winter (Figure 1A; *R*^2^ = 0.35, *p* < 0.001). Of the neonates born in winter, 85.3% had cord blood 25(OH)D concentrations <30 nmol/L, and 98.9% had concentrations <50 nmol/L, compared to 5.8 and 40.3% of neonates born in autumn, respectively (Figure 1B; *p* < 0.001).

Table 2 presents the results of the trend analyses, which indicated that the best-fit relationship was observed in the regression model using a quadratic function between the ambient temperature of the eighth GM (29–32 gestational weeks) and cord blood 25(OH)D concentration. Thus, the eighth GM was considered the critical time for maternal exposure to temperature-influencing cord blood 25(OH)D concentrations in subsequent analyses. Figure 2 shows that cord blood 25(OH)D concentrations significantly increased with increasing ambient temperature in the eighth GM. Maternal exposure to ambient temperatures of 10 and 24.5°C was linked to the cutoff for vitamin D deficiency and vitamin D inadequacy in cord blood, respectively. Supplementary Figure S1 shows the relationship between ambient temperature in the different GM and cord blood 25(OH)D levels in pregnant women. We found similar thresholds, in that maternal exposure to different ambient temperatures was linked to the cutoff of vitamin D deficiency and vitamin D inadequacy.

Compared with maternal exposure to ≥24.5°C in the eighth GM, maternal exposure to 10–24.4°C or <10°C significantly elevated the risk of vitamin D deficiency and vitamin D inadequacy in cord blood. This increased risk remained significant even after adjusting for potential covariates and vitamin D supplementation during pregnancy. Additionally, maternal vitamin D supplementation for more than 2 months was significantly associated with a reduction in the risk of vitamin D deficiency and inadequacy in cord blood, even after controlling for the potential covariates and ambient temperature in the eighth GM (Table 3).

Stratified by ambient temperature in the eighth GM, the relative contributions of temperature in the eighth GM and vitamin D supplementation during pregnancy to cord blood 25(OH)D concentration were estimated. For maternal exposure to <10°C in the eighth GM, higher ambient temperature failed to significantly enhance cord blood 25(OH)D concentrations and only accounted for 0.3% of the variance. However, vitamin D supplementation was significantly associated with higher 25(OH)D concentrations in the cord blood and accounted for 0.5% of the variance. For maternal exposure to 10–24.4°C in the eighth GM, the temperature accounted for 11.7% of the variance in cord blood 25(OH)D concentrations, and vitamin D supplementation accounted for an additional 13.8% of the variance. For maternal exposure to ≥24.5°C in the eighth GM, the temperature accounted for 9.5% of the variance in cord blood 25(OH)D concentrations; however, vitamin D supplementation during pregnancy failed to significantly enhance cord blood 25(OH)D concentrations and only accounted for 0.4% of the variance.

## Discussion

In this study, ambient temperature in the eighth GM was suggested as an effective predictor of vitamin D status in full-term neonates. The mean 25(OH)D concentration in cord blood significantly increased with increasing ambient temperature in the eighth GM. Maternal exposure to 10–24.4°C in the eighth GM had a significantly higher risk of vitamin D deficiency, and the risk was further substantially elevated when the mother experienced ambient temperature < 10°C in the eighth GM, even after controlling for potential covariates and vitamin D supplementation during pregnancy. Furthermore, an interesting finding was that the necessity of vitamin D supplementation during pregnancy appeared to be dependent on ambient temperature in the eighth GM. For maternal exposure to ≥24.5°C in the eighth GM, vitamin D supplementation during pregnancy failed to significantly enhance neonatal vitamin D concentrations. In contrast, for maternal exposure to <10°C in the eighth GM, maternal vitamin D supplementation may be a unique and effective way to increase the neonatal vitamin D concentration. To the best of our knowledge, this is the first study to focus on the relationship between maternal exposure to ambient temperature and fetal vitamin D concentrations.

Our study provides important evidence that ambient temperature can serve as a useful predictor of fetal vitamin D status and suggests that maternal vitamin D supplementation should be tailored to seasonal changes in UVB exposure. Our study provides actionable recommendations for public health policies. First, temperature-based predictors are integrated into clinical guidelines for vitamin D supplementation, especially in regions where the ambient temperature falls below the critical threshold. Second, we created geographically and seasonally tailored policies for vitamin D supplementation with an emphasis on high-risk groups. Third, supporting public health campaigns and maternal health surveillance focused on the risk of seasonal vitamin D deficiency. Fourth, routine vitamin D screening is based on ambient temperature data, especially for pregnant women during winter months. Vitamin D supplementation is essential for maintaining maternal and fetal health (20), particularly to prevent complications, such as gestational diabetes mellitus (GDM) and preeclampsia (21, 22). Temperature-based adjustments to supplementation may be a practical strategy to ensure optimal vitamin D levels, particularly in regions with low sunlight exposure. Previous studies have shown that temperature changes increase the risk of GDM and preeclampsia (23, 24). However, the direct effects of temperature-based vitamin D supplementation on these outcomes have not yet been fully explored. Further studies are required to confirm the efficacy of temperature-guided supplementation for reducing pregnancy-related complications.

Vitamin D is synthesized in the skin through exposure to UVB radiation, and solar UVB radiation is the primary source of vitamin D for most humans. Therefore, the assessment of vitamin D status in populations must consider sunlight exposure (15). However, sunlight is a complex exposure to measure because ambient UVB concentrations vary up to 30-fold by latitude, season, ground surface, time of day, clothing, sunscreen, and cloud or tree cover (25–29). Thus, simply asking people how much time they spend outside may not correctly classify UVB exposures. Studies assessing the relationship between sunlight measures and serum 25(OH)D levels also showed low correlations (30, 31); sunlight measures only accounted for approximately 10% of the variation in serum 25(OH)D concentrations. The ambient temperature is easy for an individual to access for an individual. Although there was no direct causality, the predictive value of ambient temperature in this study was substantial because the ambient temperature in the eighth GM at ≥10°C could account for approximately 12% of the variation in infant 25(OH)D concentrations, which appears to be more important than the season (32) and is similar to sunlight. In addition, ambient temperature can serve as a useful indirect measure, because temperature and UVB radiation levels are often positively correlated. For example, UVB radiation tends to be higher during warmer months and at higher latitudes closer to the equator, which corresponds to higher ambient temperatures. Previous studies (33, 34) have demonstrated that the UVB radiation intensity is associated with seasonal temperature changes. We acknowledge that ambient temperature alone is an imperfect proxy but is often useful when combined with other contextual data (e.g., seasonality or geographic latitude).

It is important to note that there was a two-month lag effect of maternal exposure to ambient temperature on fetal vitamin D concentrations. The complex progression of the vitamin D endocrine system (35) and placental transmission of 25(OH)D (36) may explain this lag effect. The lag effect could be linked to the fact that maternal vitamin D synthesis is less effective during the winter months because of lower sunlight exposure, especially when ambient temperatures are low (e.g., <10°C). This reduced synthesis may take time to manifest as a deficiency or insufficiency in both the mother and the fetus. Another possible mechanism is placental transmission of 25(OH)D. Research suggests that the placenta is a key mediator of vitamin D transfer (37); however, the efficiency of this process can be affected by several factors, including maternal vitamin D status and ambient temperature. Placental transfer of vitamin D is not instantaneous and may involve a temporal delay, especially when maternal vitamin D levels are low. If the lag effect is further confirmed in the whole pregnancy through animal study, it will give an important “time window” to intervene ahead of time, which was supposed to start from 2 months before pregnancy. Furthermore, the findings of this study suggest that when pregnant women experience an ambient temperature of less than 10°C, vitamin D supplementation may be a unique and effective way to increase fetal vitamin D concentrations, as little detectable production of precholecalciferol occurs in the season of low ambient temperature when there are few ultraviolet photons with energies between 290 and 315 nm (38). This result was supported by a study from Australia showing that the contribution of sunlight to 25(OH)D concentrations is very important in the summer months but less important in the winter months (39). Given the adverse pregnancy outcomes associated with vitamin D deficiency, the potential public health implications of our findings are of immense importance.

In our study, we did not find a significant association between pre-pregnancy BMI or parity and fetal vitamin D levels (measured using cord blood 25(OH)D concentrations). This result contrasts with previous studies that have suggested that these maternal factors may influence vitamin D status (40, 41) during pregnancy. This may be because of several reasons. First, it was conducted in Hefei, China, a region that may have different seasonal patterns of UVB exposure and lifestyle factors. Second, in our cohort, maternal vitamin D supplementation was likely more widespread, especially in regions with lower UVB radiation, potentially reducing the impact of maternal BMI or parity on fetal vitamin D levels.

Our study has two major strengths. First, this is the first ecological study on the relationship between maternal exposure to gestational ambient temperature and fetal vitamin D status. Second, in contrast to previous ecological studies, adjustment for individual information (including GWG, pregnancy complications, paternal alcohol consumption, and infant sex) strengthened the conclusions of this study. Third, we considered the timing of gestational months when analyzing the effects of ambient temperature on fetal vitamin D status. This stratification allowed us to capture more nuanced relationships, which may have been overlooked in studies that did not account for such temporal factors.

However, this study has several limitations that should also be acknowledged. The missing data from October to December is an important limitation that may affect the ability of the study to fully capture the seasonal variations in ambient temperature and fetal vitamin D levels. The absence of this key period could lead to underestimation or overestimation of seasonal effects, especially because these months typically represent the transition from fall to winter, when temperature and sunlight levels fluctuate more dramatically. In addition, our study had a short duration, and the results were preliminary. Another limitation was that the duration of supplementation, but not the dose, was used to estimate maternal vitamin D supplementation in this study. Although the dose of commercial vitamin D3 that most pregnant women consumed ranged from 300 to 600 IU/d, the absence of an exact dose might have confounded the contribution of vitamin D supplementation in our study. Without knowing the exact dosage, it is challenging to determine the precise contribution of supplementation to vitamin D levels observed in cord blood. Additionally, we acknowledge that relying on self-reported data on vitamin D supplementation, including the duration of supplementation, introduces the possibility of recall bias. This bias occurs when participants may have forgotten, misremembered, or exaggerated the extent of their supplementation. Recall bias is a common challenge in retrospective studies, especially when assessing dietary or health behaviors over long periods. Although we did not capture the exact dosages, the range of supplementation (300–600 IU/day) was relatively narrow, and this range is consistent with the general guidelines for pregnant women. Overall exposure to vitamin D supplementation was within a reasonable range for most participants. In addition, a previous study (5) successfully used self-reported supplementation duration as a proxy for the exact dose of vitamin D supplementation in pregnant women, particularly when the exact doses are difficult to capture. Third, our study was based on a single cohort from Hefei, China, which may limit the generalizability of our findings to other regions with differing latitudes, UVB exposures, and socioeconomic conditions. Therefore, the results are geographically specific, and further multicenter or multi-region studies are needed to validate the relationship between ambient temperature and neonatal vitamin D levels. This would help identify whether ambient temperature during gestation is a universal predictor of fetal vitamin D status or whether it varies according to regional factors such as UVB exposure, diet, and cultural practices regarding sunlight exposure. Fourth, data collection from 2008 may not fully reflect current conditions such as climate change, advancements in supplementation practices, or shifts in maternal behaviors. Rising temperatures and shifting seasonal patterns may affect the amount of UVB exposure and, in turn, the vitamin D status of the pregnant women. Additionally, advancements in vitamin D supplementation practices (42), which have become more standardized over the past decade, could have influenced our results. Public health campaigns promoting prenatal vitamin D supplementation may increase compliance among pregnant women, potentially altering the relationship between ambient temperature and vitamin D levels. Despite the potential limitations of using historical data, these findings can provide valuable insights into the fundamental relationship between ambient temperature and vitamin D levels during pregnancy. Fifth, while the *R*^2^ value is significant, it does not explain a large proportion of the variance in neonatal vitamin D levels. This is an important point, as it suggests that there may be additional factors contributing to the variability in the data that are not captured by the ambient temperature alone. We suggest that the inclusion of other uncollected covariates could improve the model’s predictive power.

## Conclusion

Maternal exposure to ambient temperature below 10°C during pregnancy is a critical predictor of vitamin D deficiency in newborns. These findings suggest that ambient temperature can be used as an indicator to guide vitamin D supplementation. Further studies are needed to confirm the predictive value of temperature and to optimize supplementation protocols based on environmental conditions.

## Data Availability

The raw data supporting the conclusions of this article will be made available by the authors without undue reservation.

## Footnotes

^1^ http://data.cma.cn/
