# Supplementary material for: The necessity of gestational vitamin D supplementation depends on ambient temperature: concern for infant vitamin D status
Source: Front Nutr. 2025 Jan 28;12:1541427. doi: 10.3389/fnut.2025.1541427 (PMC11810728; doi:10.3389/fnut.2025.1541427)
Supplement: Supplementary file 1 [file Image_1.pdf]

Supplementary Material

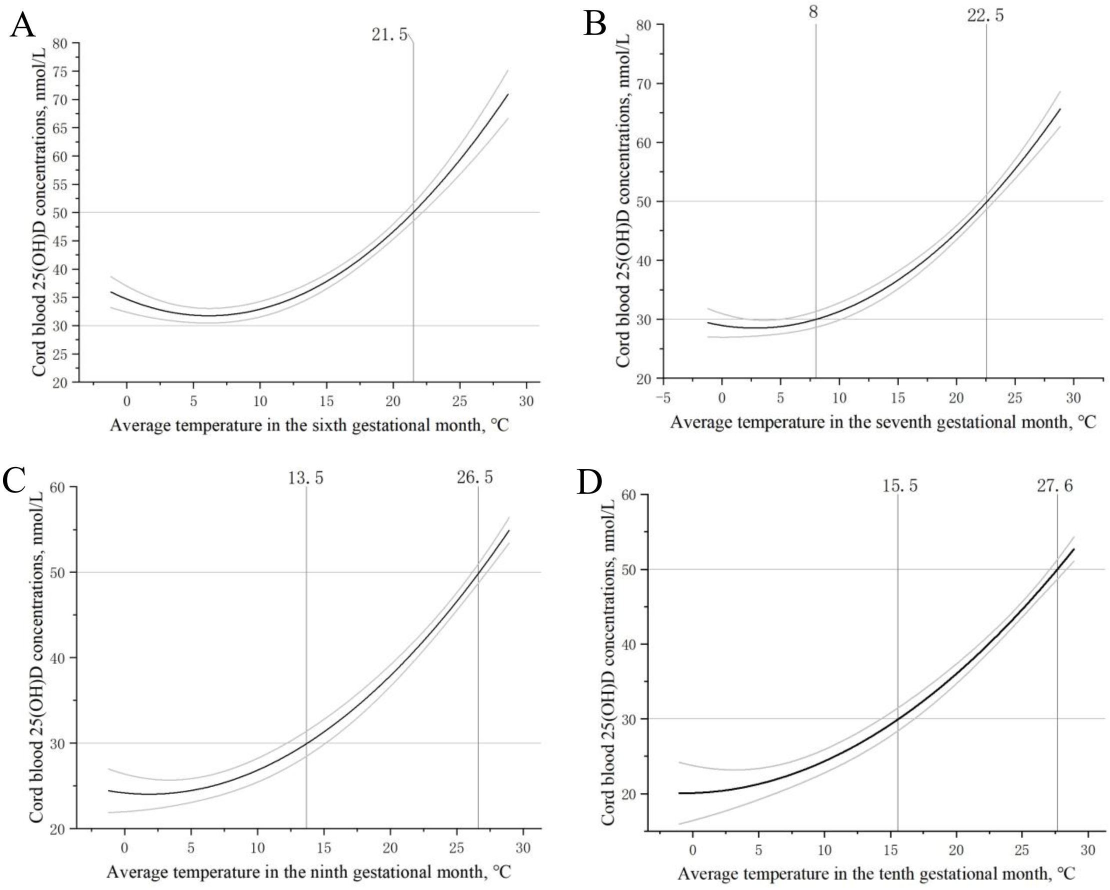

Supplementary Figure 1 Relationship between ambient temperature in the different GM and cord blood 25(OH)D in pregnant women. A: sixth GM, B: seventh GM, C: ninth GM, D: tenth GM.
The solid dark line represents the trend for changes in cord blood 25(OH)D in mean values across increasing ambient temperature in the eighth GM and grey lines represent the 95% confidence interval.
